# Challenging inhibitory control with high- and low-calorie food: A behavioural and TMS study

**DOI:** 10.3389/fnut.2023.1016017

**Published:** 2023-02-22

**Authors:** Valentina Bianco, Domenica Veniero, Alessia D’Acunto, Giacomo Koch, Silvia Picazio

**Affiliations:** ^1^Laboratory of Experimental Neuropsychophysiology, Santa Lucia Foundation IRCCS, Rome, Italy; ^2^Laboratory of Cognitive Neuroscience, Department of Languages and Literatures, Communication, Education and Society, University of Udine, Udine, Italy; ^3^School of Psychology, University of Nottingham, Nottingham, United Kingdom; ^4^Human Physiology Section, Department of Neuroscience and Rehabilitation, University of Ferrara, Ferrara, Italy; ^5^Department of Psychology, Sapienza University of Rome, Rome, Italy

**Keywords:** food, high-calorie, low-calorie, Go/NoGo, inhibitory-control, transcranial magnetic stimulation (TMS), primary motor cortex (M1)

## Abstract

Most people are often tempted by their impulses to “indulge” in high-calorie food, even if this behaviour is not consistent with their goal to control weight in the long term and might not be healthy. The outcome of this conflict is strongly dependent on inhibitory control. It has already been reported that individuals with weaker inhibitory control consume more high-calorie food, are more often unsuccessful dieters, overweight or obese compared to people with more effective inhibitory control. In the present study, we aimed at investigating inhibitory control in the context of human eating behaviour. A sample of 20 healthy normal-weight adults performed a 50% probability visual affective Go/NoGo task involving food (high- and low-calorie) and non-food images as stimuli. Single-pulse transcranial magnetic stimulation (TMS) was administered over the right primary motor cortex (M1) either 300 ms after image presentation to measure corticospinal excitability during the different stimulus categories or 300 ms after the appearance of a fixation point, as a control stimulation condition. The experimental session consisted of a food target and a non-food target block. Behavioural outcomes showed a natural implicit inclination towards high-calorie food in that participants were faster and more accurate compared to the other categories. This advantage was selectively deleted by TMS, which slowed down reaction times. MEPs did not differ according to the stimulus category, but, as expected, were bigger for Go compared to NoGo trials. Participants judged high-calorie food also as more appetising than low-calorie food images. Overall, our results point to a differential modulation when targeting inhibitory control, in favour of the more palatable food category (high-calorie). Present data suggest that the activity of the motor system is modulated by food nutritional value, being more engaged by appetising food. Future work should explore to what extent these processes are affected in patients with eating disorders and should aim to better characterise the related dynamics of cortical connectivity within the motor network.

## 1. Introduction

Eating and managing caloric intake is essential for our survival. Food-related choices mediate a large part of a person’s well-being from many points of view such as health, social life, and self-esteem. Since the dawn of neuroscience, the salience of food stimuli for the nervous system has been clearly recognised. The eminent Russian physiologist Pavlov has in fact demonstrated how food generates an unconditioned response and how other stimuli become salient if repeatedly associated with it (1).

Most people, are often tempted by their impulses to “indulge” in high-calorie food, even if this behaviour is not consistent with their goal to control weight in the long term (e.g., 2). This conflict is exacerbated by our social environment, where the abundance of appetising high-calorie food can trigger overconsuming in individuals with enhanced food cue reactivity (3). The outcome of this conflict between short-term gratification and long-term goal is strongly dependent on inhibitory control, i.e., the ability to withhold pressing responses (4). In line with this view, previous studies have shown that individuals with weaker inhibitory control consume more high-calorie food, are more often unsuccessful dieters, and are more often overweight or obese compared to people with more effective inhibitory control (5–8). In contrast, individuals with abnormal inhibitory control can exhibit a dysfunctional restriction of food intake and weight loss (9, 10).

Previous work acknowledged that reactivity to food cues is part of a trait that combines increased appetitive drive and reduced inhibitory control, which in turn would explain why some individuals are more prone to uncontrolled eating or at the opposite restrictive eating behaviour (9, 11, 12). The ability to control impulses is challenged by appetizing stimuli. This does not seem to be related to the need to procure the food necessary for the sustenance of the organism (homeostatic drive), but rather resembles a mechanism similar to addiction (hedonic drive) (13, 14), which in some cases can become very harmful (15).

A large body of research showed that inhibitory control plays a crucial role in balancing food behaviour and in the psychopathology of eating disorders (16–21).

However, studies that have investigated food-related inhibitory control in healthy participants are few and have shown inconsistencies (22).

Inhibitory control can be assessed through the Go/NoGo task. Participants are instructed to respond to a target (Go trial) and withhold response to a non-target (NoGo trial), whilst response speed and accuracy are measured. Previous studies used a classic version of this task with abstract stimuli to investigate the relationship between inhibitory control and eating behaviour (23). However, both top-down inhibitory control and bottom-up drive to food stimuli interact to determine eating behaviour (24). Therefore, Go/NoGo tasks incorporating food stimuli are likely to be more informative.

Previous studies already used this task including food stimuli with promising results, but with some limitations. For instance, Batterink et al. (25) developed a Go/NoGo task using healthy food as Go stimuli and unhealthy food images as NoGo stimuli but lacked a control stimulus, such as non-food pictures, and the sample was limited to female participants. A following study (26), measured response inhibition in food and non-food trials in males and females, but the task used words rather than images, with the undesired involvement of reading ability and abstract thought.

Although inhibitory control has been traditionally considered to rely exclusively on the prefrontal cortex, recent findings using transcranial magnetic stimulation (TMS) have shown that other areas are involved. Not only does the prefrontal cortex send its inhibitory command to the primary motor cortex (M1) but other nodes of the motor system, such as the cerebellum, play an active role in inhibitory control (27, 28).

The aim of this study was twofold. First, we wanted to study inhibitory control when different food stimuli (high-calorie vs. low-calorie) are presented in a design that would overcome previous studies’ limitations, by using images of food rather than words and by comparing the obtained response to a control stimulus (non-food). Second, we wanted to investigate the involvement of the motor system in inhibitory control and food-related environmental cues. Specifically, we used a Go/NoGo task with food and non-food images as Go and/or NoGo stimuli and concurrently investigate the excitability of the primary motor cortex (M1) collecting motor-evoked potentials (MEPs) elicited by Transcranial Magnetic Stimulation (TMS) in healthy eaters. We targeted M1 as a part of the inhibitory system because it can be easily accessed by TMS and because it provides a direct measure of the system excitability *via* MEP amplitude.

Since other factors such as current hunger or body weight may interfere with inhibitory mechanisms (22, 29), all participants were healthy eaters and they were tested under the same satiety state. Finally, we evaluated the relation between individual impulsivity traits and behavioural measures. We hypothesised that participants were faster and more accurate when presented with high-calorie, compared to low-calorie and non-food images, because they were adaptively and implicitly prompted to react at targets with good nutritive value.

## 2. Materials and methods

### 2.1. Participants

A gender-balance sample of 20 healthy participants (10 females: age 27.9 ± 3.8; years of education >13; body mass index – BMI 23.3 ± 2.9) was recruited. The sample size for the main mixed-design ANOVA (Stimulus type × TMS) was determined with G*power software (30). The effect size was estimated from a previous study (31). We set the expected effect size f(U) at 0.38, the *α* level at 0.05 and the desired power (1 − *β*) at 80%.

Inclusion criteria were the absence of any reported neurological or psychological disorders and the absence of eating disorders as measured by EDE-Q global score (32, 33). Moreover, vegetarian or vegan participants were excluded, as well as those who claimed to have particular food preferences or restrictions related to intolerances, allergies to foods or metabolic compromissions (e.g., diabetes and celiac disease). All participants were right-handed (Edinburgh Handedness Inventory, 34), reported normal or corrected-to-normal vision and were naïve about the aim of the study. Written informed consent was obtained from all participants according to the Declaration of Helsinki. The project was approved by the Santa Lucia Foundation IRCCS of Rome Ethical Committee.

### 2.2. Experimental procedure

Participants were seated in a comfortable armchair in a dimly illuminated, electrically shielded, and sound-proof room. Since fasting levels might have an impact on food related inhibitory performance (35), the experimental procedure consisted of a single session that was programmed at least 2 h after the last meal. Participants first performed the Go/NoGo task whilst TMS was delivered to the right primary motor cortex. At the end of the session, they were asked to rate the palatability of the food images presented during the task and to fill out questionnaires (see below for details).

### 2.3. Food Go/NoGo task

**Figure 1:**
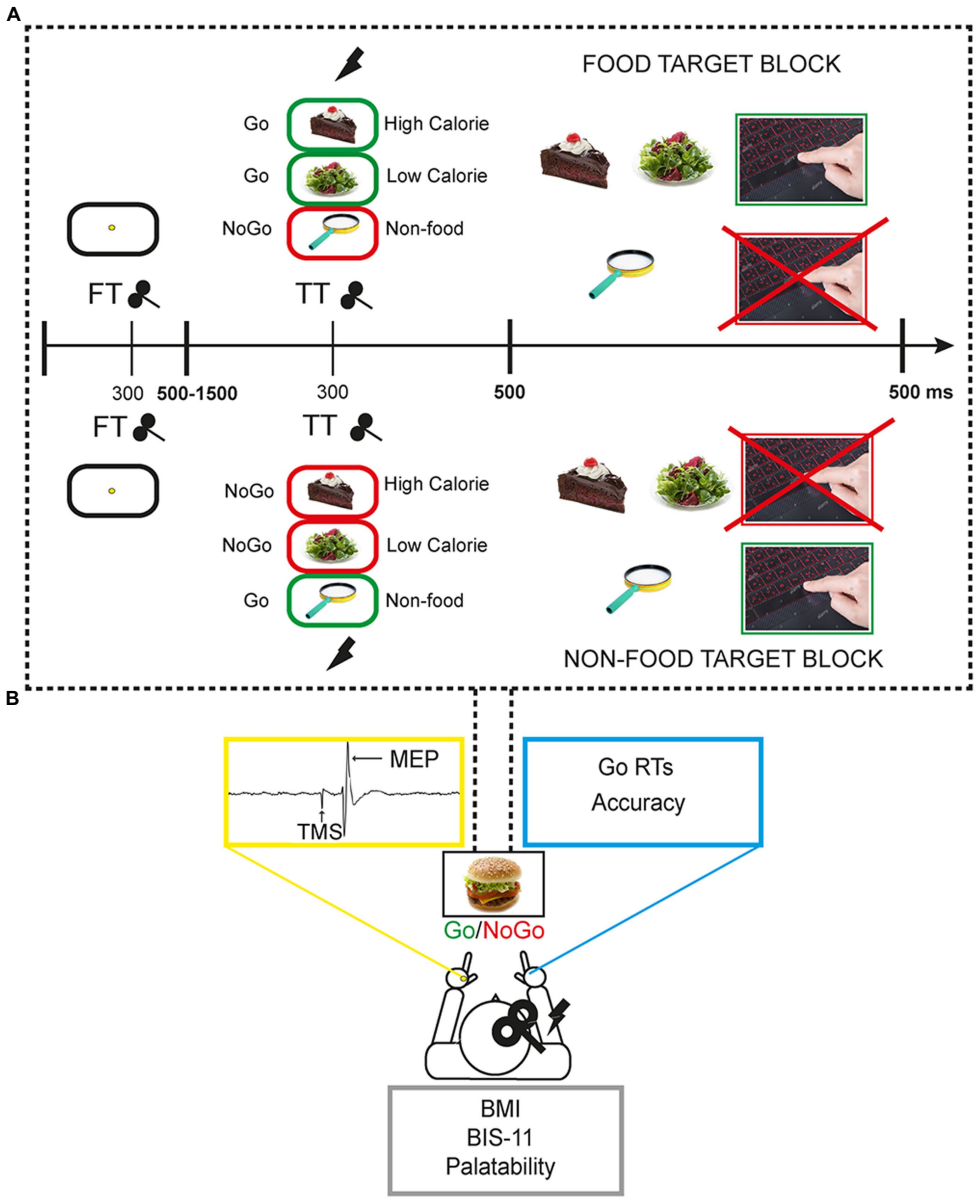
Experimental setting and behavioural task. **(A)** Each participant performed the task responding with the right hand whilst single pulse transcranial magnetic stimulation (spTMS) was applied over the right primary motor cortex (M1) and motor evoked potentials (MEP) were collected from the left hand. Each participant self-reported height and weight for BMI calculation, filled the Barratt-Impulsivity scale 11, and expressed its judgement about the palatability of task food images in a 5-point scale. **(B)** The Go/NoGo task was composed of two blocks in which participants had to alternatively respond to food or non-food stimuli.by pressing the spacebar. In FT trials spTMS was delivered 300 ms following the fixation point onset, in TT trials spTMS was delivered 300 ms following the target food/non-food target image onset. In NT trials spTMS was not delivered.

A 50% probability visual Go/NoGo task with food and non-food images was used. Images were selected from the extended food-pics database (36). Food stimuli included high-calorie and low-calorie food pictures whereas non-food images represented pleasant inedible objects. The experimental task consisted of a food-target and a non-food target block. In the food-target block, participants had to respond by pressing the space bar of a QWERTY keyboard with the right index finger when they recognised a food (Go trials) and refrain from responding when they saw a non-food picture (NoGo trials). In the non-food target block, the Go/NoGo categories were reversed (Figure 1A).

In each block, a total of 384 trials were presented, including 192 (50%) non-food images and 192 (50%) food images. Of the 192 food stimuli, 96 were high-calorie and 96 low-calorie. Each trial started with a fixation point (a yellow point at the centre of a white screen) presented for a variable interval between 500 ms and 1,500 ms (mean = 975 ms). The timing of the fixation point was varied pseudo-randomly to prevent the predictability of the stimulus. This was followed by a food or non-food image, presented for 500 ms. The duration of each trial ranged from 1,500 to 2000 ms. The duration of each block was approximately 10 min interspersed with two short breaks. Overall, the task lasted around 25 min. It is worth noting that having an equal probability of Go/NoGo trials could be less effective to elicit a clear inhibitory activity compared to Go/NoGo tasks with rare NoGo trials (37). However, the present choice was motivated by the need to have two equal blocks only differing in the instructions, and complicated by the existence of two (high-, low-calorie) food categories to be balanced with the non-food condition. Moreover, this choice was based on previous results by our group showing that a simple 50% probability Go/NoGo task can effectively modulate frontocentral cortical activity that is related to inhibitory control (Figure 1A; 28, 38).

The task was programmed and run through E-Prime 2.0 Professional software and stimuli were shown on a 23 inches monitor. For each task condition, reaction times (RTs) in ms and accuracy scores (the percentage of correct responses, i.e., button press for Go trials and no button press for NoGo trials) were collected. Both speed and accuracy were encouraged for task performance (Figure 1B).

### 2.4. Transcranial magnetic stimulation (TMS)

Single-pulse transcranial magnetic stimulation (TMS) was administered throughout the experiment over the right primary motor cortex (M1) to measure corticospinal excitability during the different task conditions (high-calorie, low-calorie, non-food). Namely, in a subset of 128 trials “TARGET TMS” (TT) [64 food (32 high-calorie, 32 low-calorie), 64 non-food stimuli] TMS was applied after 300 ms from the food/non-food picture appearance. In a subset of 128 trials “FIXATION TMS” (FT) [64 food (32 high-caloric, 32 low-caloric), 64 non-food stimuli] TMS was applied during the presentation of the fixation point, to have a control condition. TMS pulse was always released with a stimulus onset asynchrony (SOA) of 300 ms. This SOA has been previously used when a single TMS pulse was combined with the execution of a task in healthy participants (Figure 1; 39–42). Finally, in a subset of 128 “NO TMS” – NT – trials [64 food (32 high-calorie, 32 low-calorie) 64 non-food stimuli] TMS was not applied, to observe participants’ behaviour in absence of any TMS interference.

TMS was performed using a MagStim Super Rapid magnetic stimulator (Magstim Company, Whitland, Wales, United Kingdom) connected to a figure-of-eight coil with a diameter of 70 mm. The magnetic stimulus had a biphasic waveform with a pulse width of about 300 μs. The coil over M1 was always placed tangentially to the scalp at the 45° angle from the midline of the central sulcus, inducing a posterior–anterior current flow. Electromyographic (EMG) traces were recorded from the left first dorsal interosseous (FDI) muscle by using 9-mm-in-diameter surface cup electrodes. The active electrode was placed over the muscle belly and the reference electrode over the metacarpophalangeal joint of the index finger. The ground electrode was placed over the left wrist. The TMS intensity was adjusted to evoke an MEP of ~1 mV peak to peak in the relaxed FDI (43). The average TMS intensity was 65 ± 12% of the maximum stimulator output.

Responses were amplified with a Digitimer D360 amplifier through filters set at 20 Hz and 2 kHz, with a sampling rate of 5 kHz and then recorded by a computer with the use of Signal software.

The average MEP peak-to-peak amplitude was calculated for each stimulus type (high-calorie, low-calorie, non-food) and TMS TT and FT conditions. MEPs above and below 2 standard deviations of the mean were removed from the analysis (44). The left FDI relaxation during the experiment was visually monitored by the experimenter who checked both the position of the hand and the EMG traces online. Participants responded to the task with their right hand, whilst the left hand, from which the MEPs were collected, was comfortably placed on an armrest. All participants were informed prior to the start of the task that their left hand could make small involuntary movements in response to the TMS. As specified above, MEPs above the 2 standard deviations were removed from the analysis to exclude trials where the muscle was not relaxed. The number of MEPs excluded for each condition was: 10.4 ± 6.5% of High-calorie Go; 12.5 ± 11.3% of Low-calorie Go; 7.3 ± 4.5% of Non-Food Go; 5.2 ± 4.8% of High-calorie NoGo; 10.4 ± 1.8% of Low-calorie NoGo; 12 ± 7.7% of Non-Food NoGo.

MEP amplitude for each stimulus type was then normalised using the MEP obtained for the FT condition, i.e., MEP amplitude obtained in the TT condition was expressed as a percentage of the amplitude recorded in FT trials.

### 2.5. Palatability of the images

After the Go/NoGo task, all high-and low-calorie food images were presented again in random order and participants were asked to score their palatability on a five-level Likert scale, from 1 (unappetising) to 5 (very appetising).

### 2.6. Impulsivity assessment

All participants filled out the Barratt Impulsiveness Scale-11 (BIS-11), a commonly used 30-item self-report questionnaire designed to assess impulsiveness (45). All items are measured on a four-point Likert-type scale. In the scoring procedure, the items are summed and the higher scores indicate greater impulsivity (ranging between 30 and 120). A summary of the BIS-11 results is reported in Table 1.

**Table 1:** BIS-11 results.

Attention	Motor impulsiveness	Self-control	Cognitive complexity	Perseverance	Cognitive instability	Total score
9.55 ± 0.63	11.6 ± 0.61	11.95 ± 0.61	10.8 ± 0.37	6.8 ± 0.42	6.45 ± 0.39	57.15 ± 1.84

Mean ± standard deviation of BIS-11 subscale and total score.

### 2.7. Eating behaviour assessment

The body Mass Index (BMI; kg/m^2^) was calculated according to the self-reported weight and height values (46). All participants completed the latest edition of the Eating Disorder Examination Questionnaire (EDE-Q 6.0 – 32, 33). The questionnaire has been extensively studied, and its psychometric properties have been demonstrated to distinguish healthy participants from patients with eating disorders. Furthermore, the EDE-Q has shown high internal consistency in both nonclinical and clinical samples. The EDE-Q provides a global score based on four subscales (Restraint, Eating Concern, Shape Concern and Weight Concern). Participants with clinical value in EDE-Q global score were excluded from the study.

### 2.8. Statistical analysis

Two-way 3 × 3 repeated measures ANOVAs were performed for each behavioural measure of interest (reaction times and accuracy scores) with factors *Stimulus type* (High-Calorie, Low-Calorie, Non-food), *TMS* (NT, FT, and TT). A two-way repeated measure ANOVA was performed on MEP amplitude with factors *Stimulus type* (High-Calorie, Low-Calorie, and Non-food), and *Trial type* (Go vs. NoGo). Statistical analyses were performed in STATISTICA 8.0 using two-tailed alpha levels of <0.05 for defining significance. *Post-hoc* comparisons were performed by paired *t*-tests (Bonferroni corrected). The effect size was indicated as partial eta square (*η*^P^_2_). The relationship between BMI and BIS-11 total scores was also investigated using Spearman’s rho coefficient.

## 3. Results

### 3.1. Palatability

**Figure 2:**
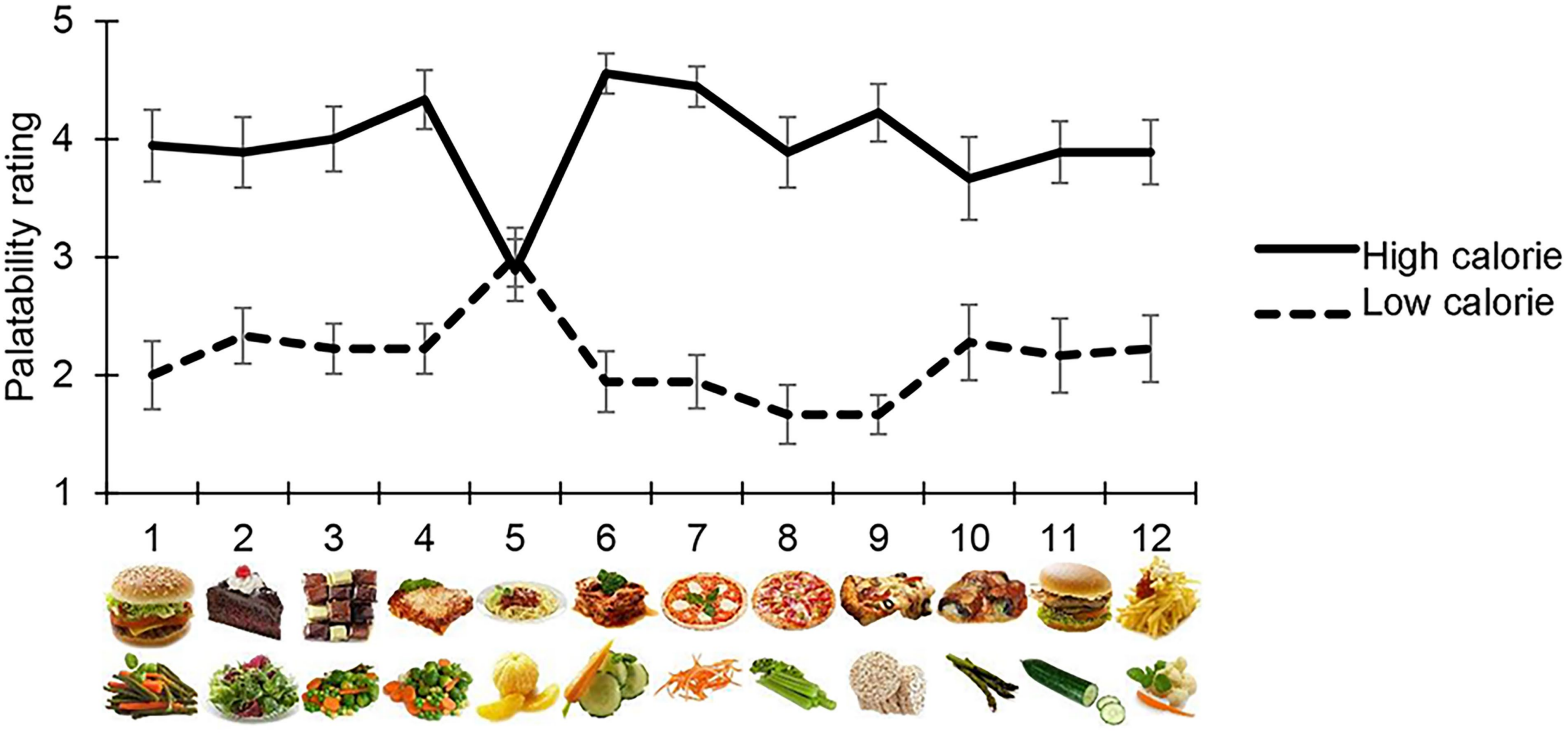
Palatability rating. Each participant judged the palatability of each food image used in the main task on a 5-point Likert’s scale. The mean palatability score is shown on the y-axis, for each high/low-calorie stimuli used (*x*-axis). With the exception of high-calorie and low calorie item 5, which were excluded from the final analysis, participants considered high-calorie food images as being more appetising than low-calorie images.

Participants judged high-calorie food as being the most appetising, with the exception of image 5 in the high-and low-calorie food categories, which were scored differently from other images of their same category, i.e., both were perceived as halfway between high-and low-palatable food (average score = 3). For this reason, all measurements collected for these images were removed from the final analysis (Figure 2).

### 3.2. Food Go/NoGo

**Figure 3:**
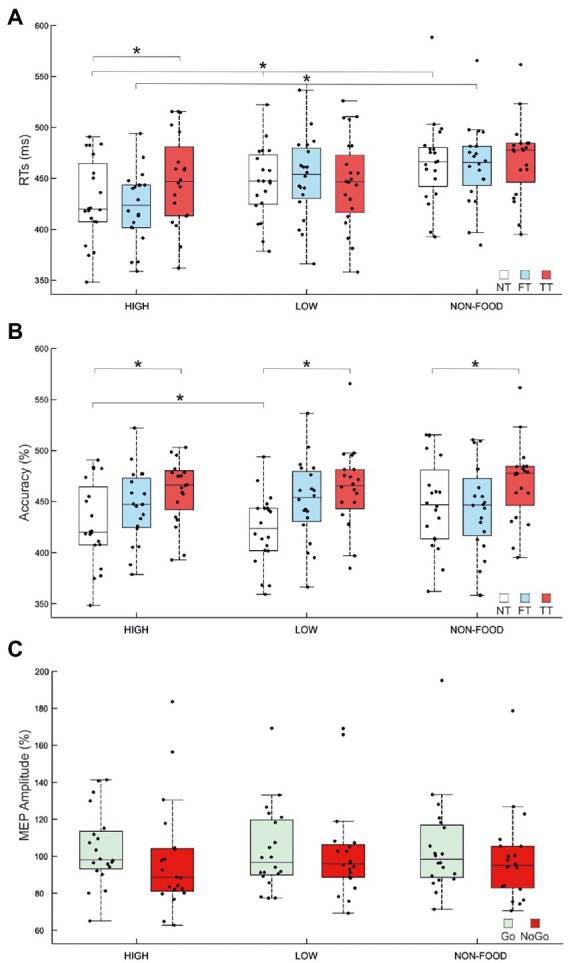
Effects of image type and TMS over MI. **(A)** Mean go reaction times (RTs) for High/Low-Calorie and Non-food trials for NT (No TMS), FT (Fixation TMS) and TT (Target TMS). In NT and FT, high-calorie RTs were faster than RTs collected for low-calorie and non-food images. Low-calorie RTs were also faster compared to non-food RTs. A specific increase of high-calorie RTs emerged between NT and TT conditions. **(B)** Mean accuracy for High/Low-Calorie and Non-food trials for NT, FT and TT. Participants were more accurate for high-calorie compared to low calorie NT trials. Accuracy decreased in TT conditions, regardless of stimulus type. **(C)** Mean normalised amplitude of motor evoked potentials (MEPs). MEP amplitude collected during the target presentation (TT) for High/Low-Calorie and Non-food trials in Go and NoGo conditions is expressed as percentage of the MEP amplitude collected at baseline (FT). As expected, corticospinal excitability is lower in NoGo conditions (*p* = 0.036). In each panel, black dots represent individual data. The box and wiskers show the Median ± 1.5 interquartile range (IQR) and asterisks represent significance.

The ANOVA on Go RTs showed significant main effects of *Stimulus type* (*F*_2,38_ = 21.44, *p* < 0.001, *ηP*_2_ = 0.53) and TMS (*F*_2,38_ = 7.86, *p* = 0.001, *ηP_2_* = 0.29) and a significant *Stimulus type* × *TMS* interaction (*F*_4,76_ = 8, *p* < 0.001, *ηP*_2_ = 0.3). We first investigated the effect of *Stimulus type* in the NT condition. *Post-hoc* comparisons indicated that, in the absence of TMS, participants were faster in response to high-calorie (mean = 428 ms) compared to low-calorie (mean = 445 ms; *p* < 0.002) and non-food images (mean = 465 ms; *p* < 0.001). Participants were also faster to respond to low-calorie food images compared to non-food images (*p* < 0.001). *Post-hoc* comparisons on the FT condition revealed the same pattern, with faster RTs to high-calorie (mean = 422 ms) compared to low-calorie food (mean = 450 ms; *p* < 0.001) and non-food images (mean = 463; *p* < 0.001). Finally, when TMS was delivered during the image presentation (TT), we found an effect specific to high-calorie food with RTs significantly slower compared to the NT condition (high-calorie TT = 448 ms vs. high-calorie NT = 428 ms; *p* < 0.001). A summary of these results is shown in Figure 3A and Tables 2, 3.

**Table 2:** Food Go/NoGo results.

	High	Low	Non-food
NT	428 ± 41 ms	446 ± 36 ms	466 ± 42 ms
	99.2 ± 1%	98.2 ± 1.9%	98.8 ± 0.9%
FT	422 ± 35 ms	451 ± 40 ms	463 ± 39 ms
	99 ± 1.3%	97.1 ± 1.9%	98.5 ± 1.4%
TT	448 ± 45 ms	444 ± 42 ms	469 ± 38 ms
	98.3 ± 1.7%	97.5 ± 2.7%	98 ± 2.2%

Mean ± standard deviation of RTs (ms) and accuracy scores (%) regarding *Stimulus type (High-, low-calorie, and non-food)* and *TMS (NT-NO TMS, FT-FIXATION TMS, and TT-TARGET TMS)* factors.

**Table 3:** RTs *post-hoc* results.

		High	Low	Non-food	High	Low	Non-food	High	Low	Non-food
		NT	NT	NT	FT	FT	FT	TT	TT	TT
High	NT		0.00259	0.00000	1	0.00003	0.00000	0.00029	0.00893	0.00000
Low	NT	0.00259		0.00029	0.00002	1	0.00328	1	1	0.00002
Non-food	NT	0.00000	0.00029		0.00000	0.02031	1	0.00259	0.00007	1
High	FT	1	0.00002	0.00000		0.00000	0.00000	0.00000	0.00007	0.00000
Low	FT	0.00003	1	0.02031	0.00000		0.15363	1	1	0.00191
Non-food	FT	0.00000	0.00328	1	0.00000	0.15363		0.02428	0.00091	1
High	TT	0.00029	1	0.00259	0.00000	1	0.02428		1	0.00020
Low	TT	0.00893	1	0.00007	0.00007	1	0.00091	1		0.00000
Non-food	TT	0.00000	0.00002	1	0.00000	0.00191	1	0.00020	0.00000	

*p* values regarding *Stimulus Type (High-, low-calorie, and non-food)* and *TMS (NT-NO TMS, FT-FIXATION TMS, and TT-TARGET TMS)* factors.

The ANOVA performed on accuracy showed significant main effects of *Stimulus type* (*F*_2,38_ = 7, *p* = 0.003, *ηP*_2_ = 0.27) and *TMS* (*F*_2,38_ = 4.4, *p* = 0.019, *ηP*_2_ = 0.19) but not significant Interaction. *Post-hoc* comparisons indicated that participants were generally more accurate to respond to high-calorie (mean = 98.84%) than low-calorie (mean = 97.59%; *p* = 0.002) and non-food images (mean = 98.43%; *p* < 0.05). We also found that TMS caused a deterioration of the accuracy in TT compared to NT conditions (*p* = 0.019), regardless of *Stimulus type*. A summary of these results is shown in Figure 3B and Tables 2, 4. No significant correlation between BIS-11 total scores and self-reported BMI was found (*R* = 0.197; *p* = 0.414).

**Table 4:** Accuracy *post-hoc* results.

TMS	NT	FT	TT	Stimulus type	High	Low	Non-food
NT		0.16	0.02	High		0.002	0.702
FT	0.16		1	Low	0.002		0.056
TT	0.02	1		Non-food	0.702	0.056	

*p* values regarding *TMS (NT-NO TMS, FT-FIXATION TMS, and TT-TARGET TMS)* and *Stimulus type (High-, low-calorie, and non-food)* factors.

### 3.3. Motor-evoked potentials (MEPs)

The repeated measure ANOVA performed to investigate changes in MEP amplitude revealed no significant main effect of *Stimulus type* (*F*_2,38_ = 0.21, *p* = 0.809), *Trial type* (*F*_1,19_ = 2.64, *p* = 0.121) and no significant Interaction (*F*_2,38_ = 0.11, *p* = 0.897). To further investigate changes in M1 excitability due to the Go/NoGo condition, we decided to run an additional ANOVA, again with factors *Stimulus type* and *Trial type*, but only considering food vs. non-food stimuli, without distinguishing between high-and low-calorie images. We found a significant main effect of *Trial type* (*F*_1,19_ = 5.129, *p* = 0.036, *ηP_2_* = 0.21), explained by a greater MEP amplitude in Go trials (mean Go amplitude = 104% vs. mean NoGo amplitude = 99%). No other significant effect was found. A summary of these results is shown in Figure 3C and Table 5.

**Table 5:** MEP results.

	High	Low	Non-food
Go	104 ± 20	103 ± 23	105 ± 27
NoGo	98 ± 30	102 ± 25	99 ± 24

Mean ± standard deviation of MEP amplitude for each *Stimulus type (High, low-calorie, and non-food)* and *Trial type (Go vs. NoGo)*. MEP in the *TT (TARGET TMS)* condition were normalised using the *FT (FIXATION TMS)* condition and expressed as a percentage.

## 4. Discussion

In the present study, we investigated whether there is a relationship between the visual appearance of food (high-calorie, low-calorie food) and inhibitory control in healthy individuals, in light of the growing interest for the topic in the eating-disorder literature (47). To this aim, we used an affective Go/NoGo task manipulating the stimulus category and coupled the behavioural measures with measures of corticospinal excitability (i.e., MEPs) to gain further insight on the contribution of the motor system. Overall, the designed task was innovative when compared to previous investigations because it used food stimuli rather than abstract stimuli (23), included a neutral condition rather than limiting the comparison to high-and low-calorie food (25) and prevented the undesired influence of reading ability or abstract thought on performance (26). Crucially, whilst previous investigations were limited to female participants (25) our sample included both normal-weighted male and female participants.

In line with a neuroimaging study using similar paradigms (48), we showed that RTs to high-calorie food were faster than RTs to low-calorie and non-food stimuli. High-calorie food images were considered also more appetising than low-calorie food images by our participants confirming the already reported correlation between the calorie content and the perceived palatability (49, 50).

This result suggests that the visual appearance of high-calorie food generates a state of heightened arousal in the observer, which in turn contributes to promptly responding to appetising food pictures and that we are naturally more inclined to respond to rewarding stimuli such as high-calorie food (48). Crucially, participants were not tested under conditions of starvation (i.e., they were invited to consume breakfast or lunch 2 or 3 h prior to the experimental session); therefore, the evidence of reduced RTs to high-calorie food was specifically powerful, even in absence of a starvation state. Collectively, these observations reinforce the view that high-calorie foods have high incentive value, prompt response (51) and increase arousal independently from satiety levels (52).

An interesting result is the increase of high-calorie RTs when a single TMS pulse was delivered over the primary motor cortex during the image presentation (TT condition). This effect is specific to high-calorie stimuli and therefore cannot be explained by a generic interference of TMS on task execution. The present result suggests that the motor system might be particularly engaged in movements aimed at approaching high-calorie and therefore high-nutritious/appetising food. In this sense, the response of the motor system, which we measure here by testing the excitability of the primary motor area, in reacting to high-calorie food compared to food with little (low-calorie) or no nutritional value (non-food) would be greater. The finding that the caloric content of food did shape task performance is particularly interesting because participants were unaware of the distinction between high-and low-calorie stimuli during the task. They were simply instructed to go or not to go in response to food or non-food images. The challenge posed by appetising foods to inhibitory control mechanisms could explain why often during a diet we cannot stop right in front of high calorie foods. Most of the attempts so far have targeted the dorsolateral prefrontal cortex (47, 53). A different strategy could target and modulate the activity of other areas of the motor circuit, such as the primary motor cortex or the cerebellum.

We also found that accuracy was increased for high-compared to low-calorie food. This result corroborates previous studies (e.g., 54) suggesting a more efficient response to foods with greater salience. It is worth noting that the accuracy reflects the ability to effectively respond as well as refrain from when a stimulus is presented. Therefore, the selection of the appropriate response (go or no go, depending on the instructions) to palatable foods might reflect a fine-tuning of cognitive control processes as a result of the increased nutritive value and the appetising nature of high-calorie food. Accordingly, several studies have found that accuracy for high-calorie food during inhibitory control tasks is reduced in overweight population (55–57), suggesting a dysregulation of the inhibitory system that is specific for palatable foods. In addition, He et al. (48) showed that the ability to inhibit response to high-calorie foods is more difficult for individuals with higher BMI and who reported to consume more high-calorie foods. However, in the present study we did not find a direct correlation between response inhibition and individual BMI or impulsivity assessment. The lack of a correlation might be due to measurement errors, since weight and height were self-reported and not objectively measured in the laboratory. Furthermore, a sample size of 20 normal-weighted participants with very low variations in BMI might not be powerful enough to unveil a possible correlation. Future studies including participants with abnormal BMI (i.e., excessively high or low) are needed to clarify this relationship.

Last, neurophysiological results (MEPs) showed an effect of trial type, with higher corticospinal excitability for Go compared to NoGo trials, independently from the stimulus category. This is in line with previous studies (58, 59) showing that the decrease in MEP amplitude is due to the inhibition of the corticospinal pathway after the NoGo decision or to the increase of corticospinal excitability following Go stimuli, in line with premovement facilitation (60). However, the present MEP results are in contrast to what already reported in a preceding TMS study showing that the additional excitatory drive triggered by salient cues counteracts the presence of inhibitory influences to M1 (61).

One possible explanation of the null result regarding the modulation of MEP amplitude according to high, low-calorie, or non-food category could be the timing of the pulse delivery, i.e., 300 ms after the image presentation which could be too late to target the dynamics of corticospinal excitability. According to this view, in a previous study, the time course of corticospinal excitability changes during a similar task found effects on MEP amplitude up to 200 ms following the onset of a simple Go/NoGo visual cue (38). In the present study, we reasoned that more complex visual stimuli (images of food or objects instead of geometric shapes), would require a longer processing time and therefore we increased the cue to TMS interval to 300 ms. However, the average RTs to the food Go/NoGo task (high: 428 ms; low: 445 ms; non-food: 465 ms) are comparable to those of the simple Go/NoGo task used in Picazio et al. (38) (428 ms). It is therefore possible that using the same cue to TMS interval in the present study could have shown differences in the corticospinal activity depending on stimulus type. Therefore, we might have missed the relevant window for MEP modulation but were still able to interfere with RTs which are the final output of the motor process involved. Another explanation of this negative result for the MEPs could be the Go/NoGo trial ratio (50/50). This could be less effective in evoking a clear motor inhibition compared to Go/NoGo tasks with rarer NoGo trials (37).

The present data do not allow us to distinguish between externally-driven action inhibition, which is typically triggered by Go/NoGo tasks and internally-driven motivational factors that here could be elicited by the affective/nutritional component of the task (62). This aspect should be explored in future studies in which participants could choose to respond or not when images are presented according to their preferences.

Finally, the present study has some limitations that could be addressed in future. First, larger sample sizes are needed to investigate any correlation between food-related inhibitory control and individual measures of body weight and impulsivity; second, subjective measures of weight and height should be replaced with objective measures; third, corticospinal excitability should be tested at different time points from stimulus presentation.

In the present study, we only tested healthy participants, but it has been previously reported that eating disorders are associated with altered responses in the control system. For example, in anorexia nervosa high-calorie food elicits an enhanced activation of cognitive control regions, explaining the persistent food avoidance and starvation (63). On the contrary, in obesity, high-calorie food is associated with abnormal activation of the impulsive system, which leads to excessive food consumption (64, 65). Therefore, in future studies should address the relationship between food-related inhibitory control mechanisms including also patients with eating disorders.

In conclusion, our findings show that the calorie content of food frequently corresponds to the perceived palatability in healthy participants and that the sight of high-calorie food triggers an implicit drive to approach and is characterised by a stronger activation of the primary motor cortex. This enhanced involvement of the motor circuit coupled with reduced reaction times and improved performance for high-calorie food might reflect the existence of adaptive mechanisms aimed to approach food with high nutritive value in healthy participants.

## Data availability statement

The raw data supporting the conclusions of this article will be made available by the authors, without undue reservation.

## Ethics statement

The studies involving human participants were reviewed and approved by Fondazione Santa Lucia IRCCS. The patients/participants provided their written informed consent to participate in this study.

## Author contributions

AD’A, SP, and VB collected the data. DV, SP, and VB analysed and interpreted the data. SP and VB wrote the paper. GK and SP designed the experiment. All authors approved the final version of the manuscript and agreed to be accountable for all aspects of the work.

## Funding

This work was supported by grant of the Italian Ministry of Health (grant number GR-2019-12369640 to SP).

## Conflict of interest

The authors declare that the research was conducted in the absence of any commercial or financial relationships that could be construed as a potential conflict of interest.

## Publisher’s note

All claims expressed in this article are solely those of the authors and do not necessarily represent those of their affiliated organizations, or those of the publisher, the editors and the reviewers. Any product that may be evaluated in this article, or claim that may be made by its manufacturer, is not guaranteed or endorsed by the publisher.

## References

[1] Pavlov IP. Conditioned Reflexes: An Investigation of the Physiological Activity of the Cerebral Cortex. Oxford: Oxford University Press (1927). 10.5214/ans.0972-7531.1017309 PMC4116985 25205891

[2] Jeffery RW Epstein LH Wilson GT Drewnowski A Stunkard AJ Wing RR. Long-term maintenance of weight loss: current status. Health Psychol. (2000) 19:5–16. doi: 10.1037/0278-6133.19.suppl1.5, PMID: 10709944

[3] Polivy J Herman CP Coelho JS. Caloric restriction in the presence of attractive food cues: external cues, eating, and weight. Physiol Behav. (2008) 94:729–33. doi: 10.1016/j.physbeh.2008.04.010, PMID: 18486161

[4] Strack F Deutsch R. Reflective and impulsive determinants of social behavior. Personal Soc Psychol Rev. (2004) 8:220–47. doi: 10.1207/s15327957pspr0803_1 15454347

[5] Jansen A Nederkoorn C van Baak L Keirse C Guerrieri R Havermans R. High-restrained eaters only overeat when they are also impulsive. Behav Res Ther. (2009) 47:105–10. doi: 10.1016/j.brat.2008.10.016, PMID: 19038379

[6] Guerrieri R Nederkoorn C Stankiewicz K Alberts H Geschwind N Martijn C. The influence of trait and induced state impulsivity on food intake in normal-weight healthy women. Appetite. (2007) 49:66–73. doi: 10.1016/j.appet.2006.11.008, PMID: 17261343

[7] Nederkoorn C Guerrieri R Havermans RC Roefs A Jansen A. The interactive effect of hunger and impulsivity on food intake and purchase in a virtual supermarket. Int J Obes. (2009) 33:905–12. doi: 10.1038/ijo.2009.98, PMID: 19546869

[8] van den Akker K Stewart K Antoniou EE Palmberg A Jansen A. Food cue reactivity, obesity, and impulsivity: are they associated? Curr Addict Rep. (2014) 1:301–8. doi: 10.1007/s40429-014-0038-3

[9] Brooks SJ O'Daly O Uher R Friederich HC Giampietro V Brammer M. Thinking about eating food activates visual cortex with reduced bilateral cerebellar activation in females with anorexia nervosa: an fMRI study. PLoS One. (2012) 7:e34000. doi: 10.1371/journal.pone.0034000, PMID: 22479499 PMC3313953

[10] Steinglass JE Figner B Berkowitz S Simpson HB Weber EU Walsh BT. Increased capacity to delay reward in anorexia nervosa. J Int Neuropsychol Soc. (2012) 18:773–80. doi: 10.1017/S1355617712000446, PMID: 22591835 PMC3638253

[11] Vainik U García-García I Dagher A. Uncontrolled eating: a unifying heritable trait linked with obesity, overeating, personality and the brain. Eur J Neurosci. (2019) 50:2430–45. doi: 10.1111/ejn.14352, PMID: 30667547

[12] Wierenga CE Ely A Bischoff-Grethe A Bailer UF Simmons AN Kaye WH. Are extremes of consumption in eating disorders related to an altered balance between reward and inhibition? Front Behav Neurosci. (2014) 8:410. doi: 10.3389/fnbeh.2014.00410, PMID: 25538579 PMC4260511

[13] Finlayson G King N Blundell J. The role of implicit wanting in relation to explicit liking and wanting for food: implications for appetite control. Appetite. (2008) 50:120–7. doi: 10.1016/j.appet.2007.06.007, PMID: 17655972

[14] Zheng H Berthoud HR. Eating for pleasure or calories. Curr Opin Pharmacol. (2007) 7:607–12. doi: 10.1016/j.coph.2007.10.011, PMID: 18037344 PMC2194753

[15] Forsén Mantilla E Clinton D Monell E Levallius J Birgegård A. Impulsivity and compulsivity as parallel mediators of emotion dysregulation in eating-related addictive-like behaviours, alcohol use, and compulsive exercise. Brain Behav. (2021) 12:e2458. doi: 10.1002/brb3.2458, PMID: 34928542 PMC8785615

[16] Bartholdy S Dalton B O'Daly OG Campbell IC Schmidt U. A systematic review of the relationship between eating, weight and inhibitory control using the stop signal task. Neurosci Biobehav Rev. (2016) 64:35–62. doi: 10.1016/j.neubiorev.2016.02.010, PMID: 26900651

[17] Juarascio AS Manasse SM Espel HM Kerrigan SG Forman EM. Could training executive function improve treatment outcomes for eating disorders? Appetite. (2015) 90:187–93. doi: 10.1016/j.appet.2015.03.013, PMID: 25777264 PMC4844012

[18] Lavagnino L Arnone D Cao B Soares JC Selvaraj S. Inhibitory control in obesity and binge eating disorder: a systematic review and meta-analysis of neurocognitive and neuroimaging studies. Neurosci Biobehav Rev. (2016a) 68:714–26. doi: 10.1016/j.neubiorev.2016.06.041, PMID: 27381956

[19] Lavagnino L Mwangi B Bauer IE Cao B Selvaraj S Prossin A. Reduced inhibitory control mediates the relationship between cortical thickness in the right superior frontal gyrus and body mass index. Neuropsychopharmacology. (2016b) 41:2275–82. doi: 10.1038/npp.2016.26, PMID: 26888057 PMC4946058

[20] Turton R Nazar BP Burgess EE Lawrence NS Cardi V Treasure J. To go or not to go: a proof of concept study testing food-specific inhibition training for women with eating and weight disorders. Eur. Eating Disord. Rev. (2018) 26:11–21. doi: 10.1002/erv.2566 29098749

[21] Yano M Kawano N Tanaka S Kohmura K Katayama H Nishioka K. Dysfunction of response inhibition in eating disorders. J Clin Exp Neuropsychol. (2016) 38:700–8. doi: 10.1080/13803395.2016.1151480 27167868

[22] Zheng L Miao M Gan Y. A systematic and meta-analytic review on the neural correlates of viewing high-and low-calorie foods among normal-weight adults. Neurosci Biobehav Rev. (2022) 138:104721. doi: 10.1016/j.neubiorev.2022.104721, PMID: 35667634

[23] Claes L Mitchell JE Vandereycken W. Out of control? Inhibition processes in eating disorders from a personality and cognitive perspective. Int J Eat Disord. (2012) 45:407–14. doi: 10.1002/eat.20966, PMID: 22006655

[24] Appelhans BM. Neurobehavioral inhibition of reward-driven feeding: implications for dieting and obesity. Obesity. (2009) 17:640–7. doi: 10.1038/oby.2008.638, PMID: 19165160

[25] Batterink L Yokum S Stice E. Body mass correlates inversely with inhibitory control in response to food among adolescent girls: an fMRI study. NeuroImage. (2010) 52:1696–703. doi: 10.1016/j.neuroimage.2010.05.059, PMID: 20510377 PMC2910204

[26] Loeber S Grosshans M Korucuoglu O Vollmert C Vollstädt-Klein S Schneider S. Impairment of inhibitory control in response to food-associated cues and attentional bias of obese participants and normal-weight controls. Int J Obes. (2012) 36:1334–9. doi: 10.1038/ijo.2011.184, PMID: 21986703

[27] Duque J Greenhouse I Labruna L Ivry RB. Physiological markers of motor inhibition during human behavior. Trends Neurosci. (2017) 40:219–36. doi: 10.1016/j.tins.2017.02.006, PMID: 28341235 PMC5389740

[28] Picazio S Ponzo V Koch G. Cerebellar control on prefrontal-motor connectivity during movement inhibition. Cerebellum. (2016) 15:680–7. doi: 10.1007/s12311-015-0731-3, PMID: 26481247

[29] Morys F García-García I Dagher A. Is obesity related to enhanced neural reactivity to visual food cues? A review and meta-analysis. Social Cognitive and Affective Neuroscience. (2020). doi: 10.1093/scan/nsaa113 [Epub ahead of print]. PMC9997070 32785578

[30] Faul F Erdfelder E Buchner A Lang AG. Statistical power analyses using G*power 3.1: tests for correlation and regression analyses. Behav Res Methods. (2009) 41:1149–60. doi: 10.3758/BRM.41.4.1149, PMID: 19897823

[31] Aulbach MB Harjunen VJ Spapé M Knittle K Haukkala A Ravaja N. No evidence of calorie-related modulation of N2 in food-related go/no-go training: a preregistered ERP study. Psychophysiology. (2020) 57:e13518. doi: 10.1111/psyp.13518, PMID: 31898816

[32] Fairburn CG Beglin SJ. Cognitive behavior therapy and eating disorders In: Fairburn CG, editor. Eating Disorder Examination Questionnaire (EDE-Q 6.0). New York: Guiford Press (2008). 309–13.

[33] Calugi S Milanese C Sartirana M El Ghoch M Sartori F Geccherle E. The eating disorder examination questionnaire: reliability and validity of the Italian version. Eat Weight Disord. (2017) 22:509–14. doi: 10.1007/s40519-016-0276-6, PMID: 27039107

[34] Oldfield RC. The assessment and analysis of handedness: the Edinburgh inventory. Neuropsychologia. (1971) 9:97–113. doi: 10.1016/0028-3932(71)90067-4 5146491

[35] Benau EM Orloff NC Janke EA Serpell L Timko CA. A systematic review of the effects of experimental fasting on cognition. Appetite. (2014) 77:52–61. doi: 10.1016/j.appet.2014.02.014, PMID: 24583414

[36] Blechert J Lender A Polk S Busch NA Ohla K. Food-Pics_Extended-an image database for experimental research on eating and appetite: additional images, normative ratings and an updated review. Front Psychol. (2019) 10:307. doi: 10.3389/fpsyg.2019.00307, PMID: 30899232 PMC6416180

[37] Wessel JR. Prepotent motor activity and inhibitory control demands in different variants of the go/no-go paradigm. Psychophysiology. (2018) 55:e12871. doi: 10.1111/psyp.12871, PMID: 28390090

[38] Picazio S Veniero D Ponzo V Caltagirone C Gross J Thut G. Prefrontal control over motor cortex cycles at beta frequency during movement inhibition. Curr Biol. (2014) 24:2940–5. doi: 10.1016/j.cub.2014.10.043, PMID: 25484293 PMC4274313

[39] Coello Y Bartolo A Amiri B Devanne H Houdayer E Derambure P. Perceiving what is reachable depends on motor representations: evidence from a transcranial magnetic stimulation study. PLoS One. (2008) 3:e2862. doi: 10.1371/journal.pone.0002862, PMID: 18682848 PMC2483935

[40] Ku Y Zhao D Hao N Hu Y Bodner M Zhou YD. Sequential roles of primary somatosensory cortex and posterior parietal cortex in tactile-visual cross-modal working memory: a single-pulse transcranial magnetic stimulation (spTMS) study. Brain Stimul. (2015) 8:88–91. doi: 10.1016/j.brs.2014.08.009, PMID: 25278428

[41] Gianelli C Kühne K Lo Presti S Mencaraglia S Dalla Volta R. Action processing in the motor system: transcranial magnetic stimulation (TMS) evidence of shared mechanisms in the visual and linguistic modalities. Brain Cogn. (2020) 139:105510. doi: 10.1016/j.bandc.2019.105510, PMID: 31923805

[42] Xia X Pi Y Xia J Li Y Shi Q Zhang J. Bilateral motor cortex functional differences in left-handed approaching-avoiding behavior. Psychophysiology. (2022) 60:e14194. doi: 10.1111/psyp.14194, PMID: 36250797

[43] Ponzo V Picazio S Di Benussi A Lorenzo F Brusa L Caltagirone C. Altered inhibitory interaction among inferior frontal and motor cortex in L-dopa induced dyskinesias. Mov Disord. (2016) 31:755–9. doi: 10.1002/mds.26520, PMID: 26861941

[44] Nguyen DTA Rissanen SM Julkunen P Kallioniemi E Karjalainen PA. Principal component regression on motor evoked potential in single-pulse transcranial magnetic stimulation. IEEE Trans Neural Syst Rehabil Eng. (2019) 27:1521–8. doi: 10.1109/TNSRE.2019.2923724, PMID: 31265402

[45] Patton JH Stanford MS Barratt ES. Factor structure of the Barratt impulsiveness scale. J Clin Psychol. (1995) 51:768–74. doi: 10.1002/1097-4679(199511)51:6<768::aid-jclp2270510607>3.0.co;2-1 8778124

[46] Oliva R Morys F Horstmann A Castiello U Begliomini C. The impulsive brain: neural underpinnings of binge eating behavior in normal-weight adults. Appetite. (2019) 136:33–49. doi: 10.1016/j.appet.2018.12.043, PMID: 30615922

[47] Costanzo F Menghini D Maritato A Castiglioni MC Mereu A Varuzza C. New treatment perspectives in adolescents with anorexia nervosa: the efficacy of non-invasive brain-directed treatment. Front Behav Neurosci. (2018) 12:133. doi: 10.3389/fnbeh.2018.00133, PMID: 30083095 PMC6064943

[48] He Q Xiao L Xue G Wong S Ames SL Schembre SM. Poor ability to resist tempting calorie rich food is linked to altered balance between neural systems involved in urge and self-control. Nutr J. (2014) 13:92. doi: 10.1186/1475-2891-13-92, PMID: 25228353 PMC4172871

[49] Drewnowski A. The role of energy density. Lipids. (2003) 38:109–15. doi: 10.1007/s11745-003-1039-3 12733741

[50] Siep N Roefs A Roebroeck A Havermans R Bonte ML Jansen A. Hunger is the best spice: an fMRI study of the effects of attention, hunger and calorie content on food reward processing in the amygdala and orbitofrontal cortex. Behav Brain Res. (2009) 198:149–58. doi: 10.1016/j.bbr.2008.10.035, PMID: 19028527

[51] Cohen D Farley TA. Eating as an automatic behavior. Prev Chronic Dis. (2008) 5:A23. 18082012 PMC2248777

[52] Kelley AE Baldo BA Pratt WE. A proposed hypothalamic–thalamic–striatal axis for the integration of energy balance, arousal, and food reward. J Comp Neurol. (2005) 493:72–85. doi: 10.1002/cne.20769, PMID: 16255002

[53] Schag K İnce B Zipfel S Max S Plewnia C Giel K. Nicht-invasive Hirnstimulation als Behandlungsverfahren bei Essstörungen – ein narrativer Überblick [Non-invasive brain stimulation in the treatment of eating disorders – a narrative review]. Psychother Psychosom Med Psychol. (2020) 70:246–51. doi: 10.1055/a-1156-8899, PMID: 32516813

[54] Carbine KA Christensen E LeCheminant JD Bailey BW Tucker LA Larson MJ. Testing food-related inhibitory control to high-and low-calorie food stimuli: electrophysiological responses to high-calorie food stimuli predict calorie and carbohydrate intake. Psychophysiology. (2017) 54:982–97. doi: 10.1111/psyp.12860, PMID: 28338210

[55] Appelhans BM Woolf K Pagoto SL Schneider KL Whited MC Liebman R. Inhibiting food reward: delay discounting, food reward sensitivity, and palatable food intake in overweight and obese women. Obesity (Silver Spring). (2011) 19:2175–82. doi: 10.1038/oby.2011.57, PMID: 21475139 PMC3303186

[56] Hall PA. Executive control resources and frequency of fatty food consumption: findings from an age-stratified community sample. Health Psychol. (2012) 31:235–41. doi: 10.1037/a0025407, PMID: 21895367

[57] Vainik U Dagher A Dubé L Fellows LK. Neurobehavioural correlates of body mass index and eating behaviours in adults: a systematic review. Neurosci Biobehav Rev. (2013) 37:279–99. doi: 10.1016/j.neubiorev.2012.11.008, PMID: 23261403 PMC4017079

[58] Leocani L Cohen LG Wassermann EM Ikoma K Hallett M. Human corticospinal excitability evaluated with transcranial magnetic stimulation during different reaction time paradigms. Brain. (2000) 123:1161–73. doi: 10.1093/brain/123.6.1161, PMID: 10825355

[59] Yamanaka K Kimura T Miyazaki M Kawashima N Nozaki D Nakazawa K. Human cortical activities during go/NoGo tasks with opposite motor control paradigms. Exp Brain Res. (2002) 142:301–7. doi: 10.1007/s00221-001-0943-2, PMID: 11819037

[60] Ficarella SC Battelli L. Motor preparation for action inhibition: a review of single pulse TMS studies using the go/NoGo paradigm. Front Psychol. (2019) 10:340. doi: 10.3389/fpsyg.2019.00340, PMID: 30846954 PMC6393403

[61] Freeman SM Aron AR. Withholding a reward-driven action: studies of the rise and fall of motor activation and the effect of cognitive depletion. J Cogn Neurosci. (2016) 28:237–51. doi: 10.1162/jocn_a_00893, PMID: 26469745 PMC5208043

[62] Ficarella SC Battelli L. The critical role of the dorsal fronto-median cortex in voluntary action inhibition: a TMS study. Brain Stimul. (2017) 10:596–603. doi: 10.1016/j.brs.2016.12.009, PMID: 28057451

[63] Scaife JC Godier LR Reinecke A Harmer CJ Park RJ. Differential activation of the frontal pole to high vs low calorie foods: the neural basis of food preference in anorexia nervosa? Psychiatry Res Neuroimaging. (2016) 258:44–53. doi: 10.1016/j.pscychresns.2016.10.004, PMID: 27866012 PMC5146322

[64] Rolls ET. Understanding the mechanisms of food intake and obesity. Obes Rev. (2007) 8:67–72. doi: 10.1111/j.1467-789X.2007.00321.x 17316305

[65] Stoeckel LE Weller RE Cook EW III Twieg DB Knowlton RC Cox JE. Widespread reward-system activation in obese women in response to pictures of high-calorie foods. NeuroImage. (2008) 41:636–47. doi: 10.1016/j.neuroimage.2008.02.031, PMID: 18413289

